# Aberrant Expression of miR-1301 in Human Cancer

**DOI:** 10.3389/fonc.2021.789626

**Published:** 2022-01-05

**Authors:** Chenming Zhong, Yiyao Dong, Qiudan Zhang, Chunhui Yuan, Shiwei Duan

**Affiliations:** ^1^Department of Clinical Medicine, Zhejiang University City College School of Medicine, Hangzhou, China; ^2^Medical Genetics Center, School of Medicine, Ningbo University, Ningbo, China; ^3^Institute of Translational Medicine, Zhejiang University City College, Hangzhou, China

**Keywords:** miR-1301, cancer, ceRNA, prognosis, signaling pathway

## Abstract

miR-1301 is a newly discovered miRNA, which is abnormally expressed in 14 types of tumors. miR-1301 inhibits 23 target genes, forms a ceRNA network with 2 circRNAs and 8 lncRNAs, and participates in 6 signaling pathways, thereby affecting tumor cell proliferation, invasion, metastasis, apoptosis, angiogenesis, etc. Abnormal expression of miR-1301 is often associated with poor prognosis of cancer patients. In addition, miR-1301 is related to the anti-tumor effect of epirubicin on osteosarcoma and imatinib on chronic myeloid leukemia(CML) and can enhance the cisplatin sensitivity of ovarian cancer. This work systematically summarizes the abnormal expression and prognostic value of miR-1301 in a variety of cancers, depicts the miR-1301-related signaling pathways and ceRNA network, and provides potential clues for future miR-1301 research.

## Introduction

There is increasing evidence that non-coding RNAs, including microRNAs (miRNAs), long non-coding RNAs (lncRNAs), and circular RNAs (circRNAs), play an important role in various biological processes (1). Long non-coding RNA (lncRNA) is an ncRNA that is more than 200 nucleotides in length. LncRNA has no coding function and can be transcribed autonomously. LncRNA has many functions, such as remodeling chromatin and genome structure, stabilizing RNA, and regulating transcription (2). Circular RNAs (circRNAs) are a new type of endogenous non-coding RNA with a closed-loop structure that can regulate linear RNA transcription, downstream gene expression, and protein production (2). MicroRNAs (miRNAs) are small non-coding RNAs (ncRNAs) of 20-22 nucleotides (3). miRNA usually binds to the 3’-untranslated regions (3’-UTRs) of its target mRNA, thereby inhibiting target gene expression (3). LncRNAs and circRNAs may competitively bind to miRNA through miRNA response elements, thereby regulating the expression level of miRNA and its target mRNA (2). Hsa-mir-1301 is a newly identified miRNA gene (4) located at chromosome 2p23.3. Hsa-mir-1301 can produce two mature miRNAs, miR-1301-3p and miR-1301-5p. At present, all miR-1301-related studies only involve miR-1301-3p, and there is no report about miR-1301-5p.

miR-1301-3p (miR-1301) is abnormally expressed in 16 cancers. Abnormal expression of miR-1301 is significantly related to the clinicopathological characteristics and prognosis of 4 cancers (4–8). miR-1301 is related to the anti-tumor effect of epirubicin (EPI) on osteosarcoma and imatinib (IM) on chronic myeloid leukemia (CML). miR-1301 also inhibit the cisplatin resistance of ovarian cancer (9–11). miR-1301 can compete with endogenous competitive RNA (ceRNA) and regulate at least 6 classic signaling pathways.

At present, many studies have shown that miR-1301 is closely related to the occurrence and development of cancer, but there is still a lack of systematic understanding of miR-1301. Therefore, we perform a PUBMED search with the following combination: “((((miR1301) OR (miR-1301)) OR (microRNA-1301)) OR (miRNA-1301)) AND (cancer)”, and conduct a comprehensive analysis of the retrieved literature. This work summarizes the diagnostic and prognostic value of miR-1301 in cancers, as well as the ceRNA network and signaling pathways it participates in and looks forward to the future challenges of miR-1301 research.

## The Aberrant Expression of miR-1301 in Cancer

**Figure 1:**
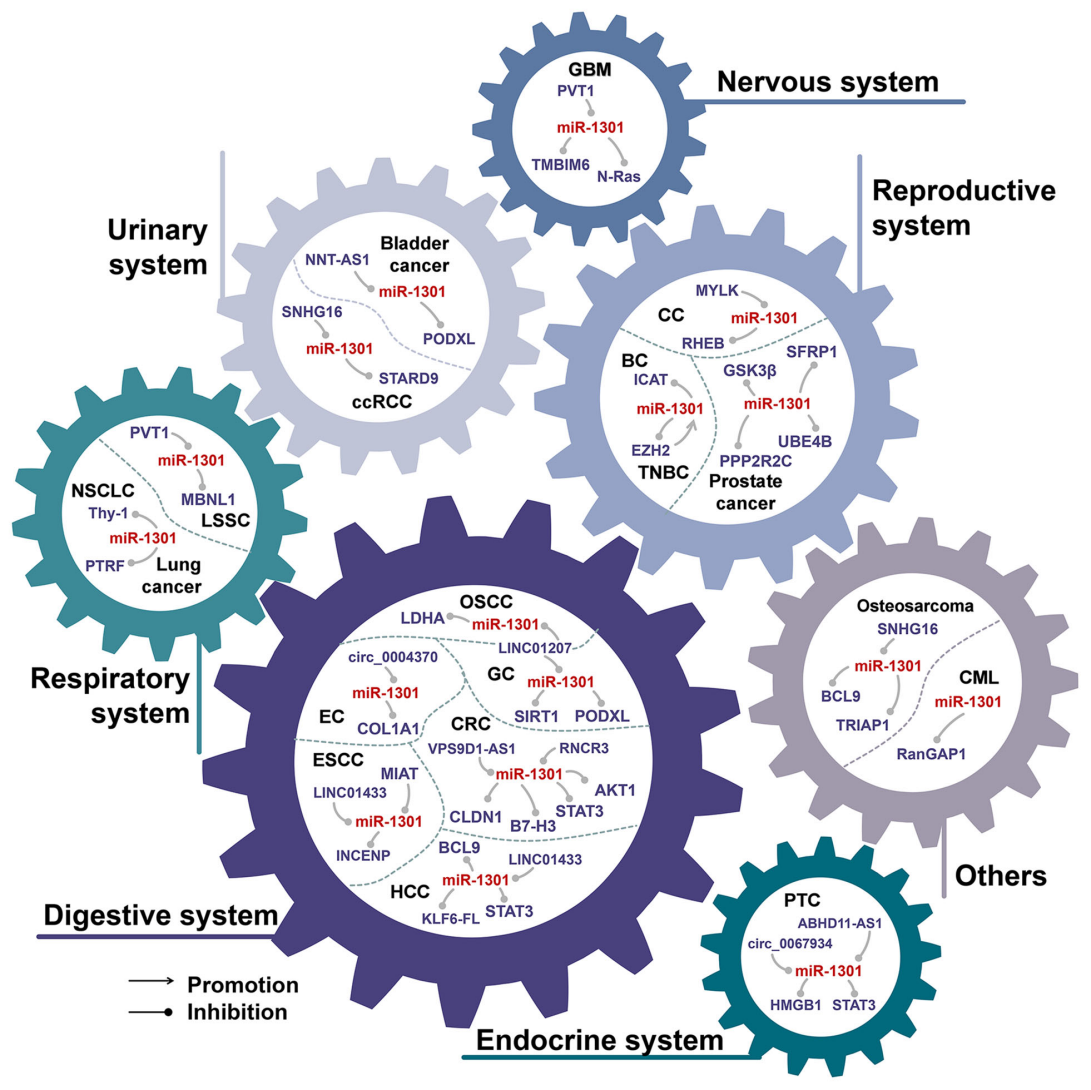
miR-1301 is abnormally expressed in 16 cancers. These diseases involve various human systems, including the digestive system, respiratory system, endocrine system, urinary system, reproductive system, nervous system, etc. EC, esophageal cancer; ESCC, esophageal squamous cell carcinoma; OSCC, oral squamous cell carcinoma; GC, gastric cancer; CRC, colorectal cancer; HCC, hepatocellular carcinoma; LSCC, Laryngeal squamous cell carcinoma; NSCLC, Non-small cell lung cancer; PTC, papillary thyroid carcinoma; ccRCC, clear cell renal cell carcinoma; CC, cervical cancer; BC, breast cancer; TNBC, triple negative breast cancer; GBM, glioblastoma multiforme; CML, chronic myeloid leukemia.

As shown in **Table 1**, miR-1301 is aberrantly expressed in 16 cancers. Among them, miR-1301 was down-regulated in 11 cancers, up-regulated in 2 cancers, and there were inconsistent expression results of miR-1301 expression in 3 cancers. miR-1301 is involved in the biological processes of tumor cell proliferation, migration, invasion, and apoptosis by regulating multiple downstream genes (**Figure 1**). In all tumor types involved in miR-1301, the abnormal expression of miR-1301 can affect the proliferation of tumor cells by affecting cell cycle and apoptosis (1, 7, 9, 12, 16, 17, 24, 26–29, 33–36). Metastasis is characterized by cancer cells invading the basement membrane into the adjacent matrix, then invading and surviving into the circulatory system (including blood and lymphatic tissue), and extravasation to distant tissues. A core step in the process of cancer metastasis is the acquisition of migratory and aggressive phenotypes (37). In 9 types of tumors, the abnormal down-regulation of miR-1301 will affect p53, WNT, STAT3, and other signaling pathways, which can promote tumor cell migration and invasion.

**Table 1:** The role of miR-1301 in various tumors.

System	Tumor type	Clinical samples	Cell lines	Animals	Expression	Regulatory mechanism	Effect *in vitro*	Effect *in vivo*	Ref.
Digestive system	EC	50 pairs of tissues (From 50 patients)	EC: OE19, KYSE410, EC109, and TE11; Normal: HEEC	nude mice (female, 4 weeks old)	Downregulation	circ_0004370/miR-1301-3p/COL1A1	proliferation↓ migration↓ invasion↓ EMT↓ apoptosis↑	tumor growth↓ (tumor volume and weight↓)	(10)
	ESCC	40 pairs of tissues (From 40 patients)	ESCC: Eca109, KYSE70, and TE-1; Normal: HEEC	–	Downregulation	LINC01433/miR-1301-3p	proliferation↓ migration↓ invasion↓	–	(11)
		–	ESCC: EC9706, EC1, EC109, and KYSE150; Normal: Het-1A	6 BALB/c nude mice (female)	Downregulation	SOX2/MIAT/miR-1301-3p/INCENP	proliferation↓ migration↓ invasion↓	tumor growth↓	(12)
	OSCC	30 pairs of tissues (From 30 patients)	OSCC: HSC-3 and HSC-4; Normal: NOK	–	Downregulation	LINC01207/miR-1301-3p/LDHA	proliferation↓ migration↓ invasion↓ apoptosis↑ autophagy↑	–	(13)
	GC	60 pairs of tissues (From 60 patients)	GC: MGC-803 and SGC-7901; Normal: GES-1	30 BALB/c nude mice (4 weeks old)	Upregulation	miR-1301-3p/SIRT1	proliferation↑ cell cycle↑	tumor growth↑ (tumor volume and weight↑)	(14)
		76 pairs of tissues (From 76 patients)	GC: MGC-803, HGC-27, MKN-45, and BGC-823; Normal: GES-1	6 BALB/c nude mice (6 weeks old)	Downregulation	CTCF/LINC01207/miR-1301-3p/PODXL	proliferation↓ migration↓ invasion↓ apoptosis↑	tumor growth↓ (tumor growth rate↓ tumor volume and weight↓)	(15)
	CRC	–	CRC: LoVo and SW480	–	Downregulation	miR-1301-3p/STAT3/MMPs	migration↓ invasion↓	–	(16)
		76 pairs of tissues (From 76 patients)	CRC: HCT116, SW480, Caco-2 and PKR Normal: NCM460	6 BALB/c nude mice (female, 5 weeks old)	Downregulation	RUNX1/RNCR3/miR-1301-3p/AKT1	proliferation↓ invasion↓ apoptosis↑	tumor growth↓ (tumor volume and weight↓)	(17)
		113 patients	–	–	Downregulation	miR-1301-3p/B7-H3	migration↓ invasion↓	tumor metastasis↓	(4)
	Colon adenocarcinoma	36 pairs of tissues (From 36 patients)	Colon adenocarcinoma: DLD1, SW620, SW480, and HCT116; Normal: FHC	–	Downregulation	VPS9D1-AS1/miR-1301-3p/CLDN1	proliferation↓ migration↓ invasion↓ apoptosis↑	–	(18)
	HCC	35 pairs of tissues (From 35 patients)	HCC: Huh-7, HepG2, Hep3B, PLC/PRF/5 and Bel-7404; Normal: MIHA	–	Upregulation	miR-1301-3p/KLF6-FL	migration↑ angiogenesis↑	–	(19)
		–	HCC: Huh-7, HepG2, Hep3B, MHCC97L, and SMCC-7721; Normal: LO2	12 BALB/c mice	Downregulation	LINC01433/miR-1301-3p/STAT3	proliferation↓ migration↓ invasion↓	tumor growth↓ (tumor volume and weight↓)	(20)
		60 pairs of tissues (From 60 patients)	HCC: Huh-7, HepG2, Hep3B, and SMMC-7721; Normal: LO2	30 BALB/c nude mice (female, 4 weeks old)	Downregulation	miR-1301-3p/BCL9	migration↓ invasion↓ angiogenesis↓ EMT↓	tumor metastasis↓	(21)
		–	HCC: HepG2; Normal: Qsg7701	–	Downregulation	–	proliferation↓ migration↓ invasion↓ apoptosis↑	–	(22)
Respiratory system	LSCC	–	LSCC: FD-LSC-1 and TU-177; Normal: HOK	–	Downregulation	PVT1/miR-1301-3p/MBNL1	proliferation↓ apoptosis↑ susceptibility to NK cells↑	–	(23)
	NSCLC	124 pairs of tissues (From 124 patients)	NSCLC: A549, H1299, MRC5, and SK-LU-1; Normal: BEAS-2B	–	Upregulation	miR-1301-3p/Thy-1	proliferation↑ migration↑ invasion↑	–	(3)
	Lung cancer	40 pairs of tissues (From 40 patients)	Lung cancer: A549 and H1299; Normal: BEAS-2B	–	Upregulation	miR-1301-3p/PTRF	proliferation↑ migration↑ EMT↑	–	(24)
Endocrine system	PTC	30 pairs of tissues (From 30 patients)	PTC: TPC-1, SW579, FTC133, MDA-T32, MDA-T120 and XTC-1; Normal: Nthy-ori3-1	–	Downregulation	circ_0067934/miR-1301-3p/HMGB1	proliferation↓ migration↓ invasion↓ EMT↓ apoptosis↑	–	(23)
		70 pairs of tissues [From 70 patients with PTC (n = 35) and benign tumors (n = 35)]	PTC: TPC-1; Normal: Nthy-ori3-1	–	Downregulation	–	proliferation↓ cell cycle↓	–	(5)
		82 pairs of tissues (From 82 patients)	PTC: BCPAP and K1; Normal: Nthy-ori 3-1	12 BALB/c nude mice (male, 4-5 weeks old)	Downregulation	ABHD11-AS1/miR-1301-3p/STAT3	proliferation↓ migration↓ invasion↓ EMT↓ apoptosis↑	tumor growth↓ (tumor volume and weight↓) tumor metastasis↓	(25)
Urinary system	ccRCC	45 pairs of tissues (From 45 patients)	ccRCC: A498, 786-O, Caki-1, and OSRC-2; Normal: HK2	–	Downregulation	SNHG16/miR-1301-3p/STARD9	proliferation↓ apoptosis↑	–	(26)
	BCa	–	BCa: RT-4, UM-UC-3, J82, and T24; Normal: SV-HUC-1	–	Downregulation	NNT-AS1/miR-1301-3p/PODXL	proliferation↓ migration↓ invasion↓ EMT↓ apoptosis↑	–	(27)
Reproductive system	CC	–	CC: DoTc2 4510, HCC94, C-33A, and HT3; Normal: Ect1/E6E7	–	Downregulation	MYLK/miR-1301-3p/RHEB	proliferation↓ apoptosis↑	–	(28)
	BC	8 pairs of tissues (From 8 patients)	BC: MCF-7, MDA-MB231, T47D, MDA-MB453, SKBR3, BT549, MDA-MB435, ZR-75-30, and Bcap37; Normal: 2 primary normal breast epithelial cells	–	Upregulation	miR-1301-3p/ICAT	proliferation↑	–	(1)
	TNBC	–	TNBC: MDA-MB231, SUM149PT, HCC1937, and HCC1806	12 nude mice (female)	Upregulation	EZH2/miR-1301-3p/EZH2	proliferation↓ migration↓ invasion↓	tumor growth↓ (tumor volume and weight↓)	(29)
	Prostate cancer	8 pairs of tissues (From 8 patients)	–	–	Upregulation	miR-1301-3p/SFRP1, GSK3β	self-renewal of PCSCs↑	–	(30)
		8 pairs of tissues (From 8 patients)	Prostate cancer: Tsu-Pr1, PC3, DU145, LNCaP, and 22RV1; Normal: prostate epithelial cells	–	Upregulation	miR-1301-3p/PPP2R2C	proliferation↑ cell cycle↑	–	(31)
		Number not shown	LNCaP and MDAPCa2b		Downregulation	miR-1301-3p/UBE4B	proliferation↓ cell cycle↓ migration↓ invasion↓ apoptosis↑	–	(32)
Nervous system	Glioma	184 patients	–	–	Downregulation	–	–	–	(2)
	GBM	6 non-cancerous brain tissues and 33 glioma tissues(low grade = 15, high grade = 18) (From 39 patients)	GBM: U87, U251, U118, LN229, A172, and H4; Normal: NHA	10 BALB/c nude mice (female, 4 weeks old)	Downregulation	miR-1301-3p/N-Ras	proliferation↓ cell cycle↓	tumor growth↓	(33)
		–	GBM: U87, U251, and T98G; Normal: NHA	nude mice (male)	Downregulation	PVT1/miR-1301-3p/TMBIM6	proliferation↓ invasion↓ apoptosis↑	tumor growth↓	(34)
Others	OS	–	OS: U2OS and SAOS-2; Normal: hFOB1.19	–	Downregulation	miR-1301-3p/TRIAP1	proliferation↓ apoptosis↑	–	(7)
		–	OS: U2OS and MG-63	–	Downregulation	SNHG16/miR-1301-3p/BCL9	proliferation↓ migration↓ invasion↓	–	(35)
		65 pairs of tissues (From 65 patients)	OS: U2OS, MG-63, SW1353, SAOS-2, and HOS; Normal: hFOB1.19	–	Downregulation	miR-1301-3p/BCL9	proliferation↓ migration↓ invasion↓	–	(6)
	CML	4 blood samples	CML: K562 and KU812; Normal: granulocytes and monocytes	–	Downregulation	miR-1301-3p/RanGAP1	–	–	(8)

EC, esophageal cancer; ESCC, esophageal squamous cell carcinoma; OSCC, oral squamous cell carcinoma; GC, gastric cancer; CRC, colorectal cancer; HCC, hepatocellular carcinoma; LSCC, Laryngeal squamous cell carcinoma; NSCLC, Non-small cell lung cancer; PTC, papillary thyroid carcinoma; ccRCC, clear cell renal cell carcinoma; CC, cervical cancer; BC, breast cancer; TNBC, triple negative breast cancer; GBM, glioblastoma multiforme; OS, osteosarcoma; CML, chronic myeloid leukemia.

### miR-1301 Is Down-Regulated in 11 Tumors

Studies have shown that miR-1301 is down-regulated in 11 cancers. These tumors include esophageal cancer (12, 38, 39), oral squamous cell carcinoma (OSCC) (13), colorectal cancer (CRC) (6, 17, 18, 40), laryngeal squamous cell carcinoma (LSCC) (41), papillary thyroid carcinoma (PTC) (7, 23, 42), clear cell renal cell carcinoma (ccRCC) (27), bladder cancer (28), cervical cancer (33), glioma (4, 24, 29), osteosarcoma (8, 9, 43), and CML (10). In these tumors, miR-1301 is regulated by at least 12 ncRNAs, including 3 circRNAs (MYLK, circ_0004370 and circ_0067934) and 9 lncRNAs (LINC01433, MIAT, LINC01207, RNCR3, VPS9D1-AS1, ABHD11-AS1, SNHG16, NNT-AS1, PVT1). Up-regulation of these upstream ncRNAs can inhibit the function of miR-1301, thereby changing the behavioral characteristics of tumor cells.

### miR-1301 Is Up-Regulated in 2 Types of Tumors

miRNA can target different downstream genes, and play an anti-cancer effect or carcinogenic effect in different tumors. Although miR-1301 is down-regulated in 9 tumors, miR-1301 is up-regulated in lung cancer (5, 21) and breast cancer (3, 20).

In four lung cancer cell lines, the expression of miR-1301 was higher than that of human normal lung epithelial cells (BEAS-2B) (5, 21). Thy-1 is a cell surface glycoprotein, which is closely related to idiopathic pulmonary fibrosis (22) and lung cancer (5). Polymerase I and transcript release factor (PTRF) is also known as cavin-1, and its down-regulation has been shown to promote the progression of prostate cancer, breast cancer, and glioblastoma (30). In lung cancer, miR-1301 acts as an oncogene by inhibiting the Thy-1 and PTRF genes (5, 21).

The expression of miR-1301 is significantly up-regulated in a variety of breast cancer cell lines and tissues, and it promotes cancer cell proliferation by directly targeting and inhibiting the expression of ICAT (3). In triple-negative breast cancer (TNBC), there is a negative feedback loop between EZH2 and miR-1301. Specifically, EZH2 can promote the expression of miR-1301, thereby inhibiting the expression of EZH2 itself. At the same time, the overexpression of miR-1301 can inhibit the proliferation of TNBC cells and the growth of xenograft tumors in mouse (20). The above evidence indicates that the dual role of miR-1301 may depend on the regulation of upstream factors in different pathological tissues. Compared with 2 primary normal breast epithelial cell lines, miR-1301 is highly expressed in 9 breast cancer cell lines. Inhibitor of β-catenin and TCF4 (ICAT) can suppress the activity of Wnt/β-catenin and play a tumor suppressor effect in a variety of tumors (31, 32). miR-1301 can inhibit the expression of ICAT, thereby increasing the risk of breast cancer (3). In addition, analysis of TCGA data showed that miR-1301 is highly expressed in TNBC. Both *in vivo* and *in vitro* experiments have shown that miR-1301 overexpression can inhibit breast cancer tumor growth (20). The tumor suppressor ability of miR-1301 may be derived from EZH2, which is a recognized oncogene (14). EZH2 is overexpressed in a variety of human malignancies (14). The overexpression of EZH2 is often associated with the advanced stage and poor prognosis of human cancer (15). It is worth noting that the expression of miR-1301 and EZH2 in TNBC is positively correlated, and the high expression of the two is correlated with poor metastasis-free survival and poor overall survival of TNBC patients (20).

### Inconsistent Results of miR-1301 Expression in 3 Kinds of Tumors

As shown in **Table 2**, there are inconsistent results of miR-1301 expression in hepatocellular carcinoma (19, 36, 44), prostate cancer (34, 35, 45), and gastric cancer (1, 16).

**Table 2:** miR-1301 is inconsistently expressed in three cancers.

Tumor type	Samples		Cell lines		Animals	Expression	Related genes		Effect *in vitro*	Effect *in vivo*	Ref.
	Dataset	Clinical samples	Tumor cell lines	Normal cell lines			Upstream genes	Downstream genes			
HCC	–	35 pairs of tissues	HCC: Huh-7, HepG2, Hep3B, PLC/PRF/5, and Bel-7404	Normal: MIHA	–	Upregulation	–	KLF6-FL (Target gene)	migration↑ angiogenesis↑	–	(19)
	–	–	HCC: Huh-7, HepG2, Hep3B, MHCC97L, and SMCC-7721	Normal: LO2	12 BALB/c mice	Downregulation	LINC01433	STAT3 (Target gene)	proliferation↓ migration↓ invasion↓	tumor growth↓ (tumor volume and weight↓)	(20)
	–	60 pairs of tissues	HCC: Huh-7, HepG2, Hep3B, and SMMC-7721	Normal: LO2	30 BALB/c nude mice (female, 4 weeks old)	Downregulation	–	BCL9 (Target gene), E-cadherin, Vimentin, Slug, and VEGF-A	migration↓ invasion↓ angiogenesis↓ EMT↓	tumor metastasis↓	(21)
	–	–	HCC: HepG2	Normal: Qsg7701	–	Downregulation	–	p53, Bcl-2, and Bcl-xL	proliferation↓ migration↓ invasion↓ apoptosis↑	–	(22)
Prostate cancer	22 normal prostate tissues and 136 prostate cancer tissues (GSE24279)	8 pairs of tissues	–	–	–	Upregulation	–	SFRP1 (Target gene), GSK3β (Target gene), OCT4, SOX2, NANOG, CD44, KLF4, MYC, and MMP2	self-renewal of PCSCs↑	–	(30)
	51 normal prostate tissues and 407 prostate cancer tissues (TCGA)	8 pairs of tissues	Prostate cancer: Tsu-Pr1, PC3, DU145, LNCAP, and 22RV1	Normal: prostate epithelial cells	–	Upregulation	–	PPP2R2C (Target gene), p27, and Cyclin D1	proliferation↑ cell cycle↑	–	(31)
	–	Number not shown	–	–	–	Downregulation	–	UBE4B (Target gene)	proliferation↓ cell cycle↓ migration↓ invasion↓ apoptosis↑	–	(32)
GC	15 normal tissues and 446 GC tissues (TCGA)	60 pairs of tissues	GC: MGC-803 and SGC-7901	Normal: GES-1	30 BALB/c nude mice (4 weeks old)	Upregulation	–	SIRT1 (Target gene), Cyclin D1, CDK4, MYC, and P21	proliferation↑ cell cycle↑	tumor growth↑ (tumor volume and weight↑)	(14)
	–	76 pairs of tissues	GC: MGC-803, HGC-27, MKN-45, and BGC-823	Normal: GES-1	6 BALB/c nude mice (6 weeks old)	Downregulation	CTCF/LINC01207	PODXL (Target gene)	proliferation↓ migration↓ invasion↓ apoptosis↑	tumor growth↓ (tumor growth rate↓ tumor volume and weight↓)	(15)

HCC, hepatocellular carcinoma; GC, gastric cancer; TCGA, The Cancer Genome Atlas.

The expression of miR-1301 in the five hepatoma cell lines (Hep3B, Huh-7, HepG2, SMMC-7721, and MHCC97L) was significantly lower than that in LO2, a human fetal hepatocyte line (19, 44). In addition, in hepatoma cell line HepG2, the expression of miR-1301 was significantly lower than that in human hepatocyte Qsg-7701 (36). However, compared with normal hepatocyte MIHA, miR-1301 has a significantly higher expression in five hepatoma cells (Hep3B, Huh-7, HepG2, Bel-7404, and PLC/PRF/5) (46).

The expression of miR-1301 in 5 prostate cancer cell lines (Tsu-Pr1, PC3, DU145, LNCAP, and 22RV1) was higher than that of normal prostate epithelial cells (34). Similarly, the expression of miR-1301 in prostate cancer tissues was also significantly higher than that in adjacent non-tumor tissues (34, 45). However, another study found that the expression of miR-1301 in prostate cancer tissues was significantly lower than that in non-cancerous tissues (35).

SIRT1 is a type III histone deacetylase that relies on nicotinamide and can participate in tumor development (25). The expression levels of miR-1301 in gastric cancer cell lines (MGC-803 and SGC-7901) are higher than that of normal human gastric mucosal cell line GES-1. miR-1301 can inhibit SIRT1, thereby exerting carcinogenic effects (16). However, another study showed that the expression of miR-1301 in gastric cancer cell lines (MGC-803, HGC-27, MKN-45, and BGC-823) was lower than that of normal human gastric mucosal cell line GES-1 (1). And this result might be due to the high expression of CCCTC binding factor (CTCF) in gastric cancer, which can activate the up-regulation of LINC01207 expression, thereby sponge miR-1301 (1).

The inconsistent results of miR-1301 expression in hepatocellular carcinoma, prostate cancer, and gastric cancer may be due to the use of different control cell lines and the presence of tissue-specific upstream regulatory factors of miR-1301. The three studies on hepatocellular carcinoma used LO2 (19, 44), Qsg7701 (36), MIHA (46) as normal control cell lines, and the hepatocellular carcinoma cell lines used in the three studies were not the same, and this may explain the inconsistent results in these three studies. The abnormal expression of miR-1301 may be closely related to upstream regulatory factors, histone deacetylation, and promoter methylation. The ceRNAs of miR-1301 comprise at least 3 circRNAs and 9 lncRNAs. In addition, EZH2 can positively regulate miR-1301 and become a target gene of miR-1301 to form a negative feedback loop. 5-Azacytidine and Trichostatin A can inhibit DNA methyltransferase (DNMT) and histone deacetylase (HDAC), respectively. Trichostatin A can significantly inhibit the expression of miR-1301 in hepatocellular cell lines (Huh7, HLC/PRF/5, and Hep3B), while 5-Azacytidine can reduce the expression of miR-1301 in Huh7 and Hep3B cell lines (46). The low expression of miR-1301 in prostate cancer cells (LNCaP, MCF-7, and HepG2) is significantly related to the hypermethylated promoter of miR-1301 (35).

## The Prognostic Value of miR-1301 in Cancer

miR-1301 is highly expressed in non-small cell lung cancer but is low expressed in glioma (4, 5), CRC (6), PTC (7), and osteosarcoma (8). In the 5 tumors, the abnormal expression of miR-1301 is closely related to tumor staging and metastasis and can lead to a poor prognosis for patients with NSCLC, glioma, and osteosarcoma (**Table 3**).

**Table 3:** The prognostic and diagnostic value of miR-1301.

Tumor type	Expression pattern	Sample size	Prognostic value	Prognosis of patients with high/low miR-1301 level	Diagnostic value	Clinicopathological characteristics	Ref.
NSCLC	Upregulation	124 patients	Independent prognostic factor for the overall survival	Poor	–	Positively related to the advanced TNM stages and lymph node metastasis	(3)
PTC	Downregulation	70 patients	–	–	sensitivity = 59.3% specificity = 98.1%	Negatively related to the advanced T and N stages	(5)
CRC	Downregulation	113 patients	–	–	–	Negatively related to the advanced TNM stages and distant metastasis	(4)
Glioma	Downregulation	184 patients	Independent prognostic factor for the overall survival and disease-specific survival	Poor	–	Negatively related to the high WHO grade, low KPS, and large tumor size	(2)
OS	Downregulation	65 patients	Prognostic factor for the overall survival	Poor	–	Negatively related to the advanced TNM stages and distant metastasis	(6)

NSCLC, Non-small cell lung cancer; PTC, papillary thyroid carcinoma; CRC, colorectal cancer; OS, osteosarcoma.

In NSCLC, the high expression of miR-1301 is significantly related to the shorter overall survival time of NSCLC patients and is also closely related to the advanced TNM stage of NSCLC and positive lymph node metastasis (5). In contrast, the expression of miR-1301 is decreased in gliomas, and the low expression of miR-1301 is significantly related to the high WHO grade, low KPS, and large tumor size of gliomas. Down-regulation of miR-1301 is also related to the decrease in the overall survival rate and disease-specific survival of glioma patients (4). In addition, the expression of miR-1301 in stage-III CRC tissue is lower than that in stage-IV CRC. In addition, the expression of miR-1301 in the serum of patients with metastatic CRC was also significantly lower than that of patients with non-metastatic CRC (6). The expression of miR-1301 decreases significantly in normal thyroid tissue, benign thyroid tumor tissue, and PTC tissue. Compared with T1-stage and N0-stage PTC tumors, miR-1301 in T2-T4-stage and N1-stage PTC tumors were decreased significantly, respectively (7). The expression of miR-1301 in osteosarcoma tissues was significantly lower than that in non-tumor tissues. The low expression of miR-1301 is significantly related to the patient’s advanced clinical stage and distant metastasis, and the patient’s poor overall survival rate (8).

## miR-1301 and the Treatment of Cancer Drugs

**Figure 2:**
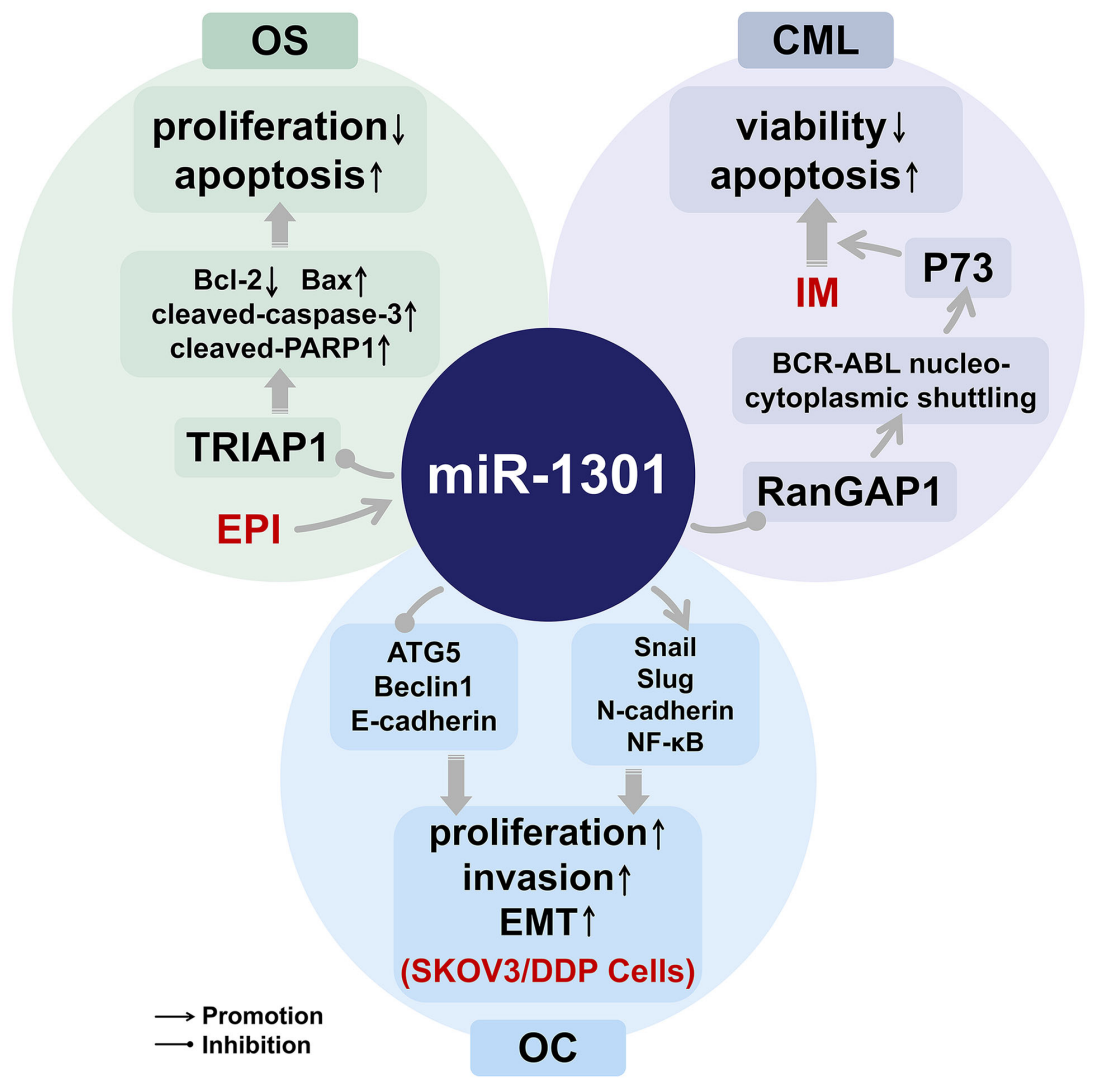
The role of miR-1301 in cancer drugs. EPI, Epirubicin; OS, osteosarcoma; IM, Imatinib; CML, Chronic myeloid leukemia; SKOV3/DDP Cells, cisplatin-resistant strain cells; OC, ovarian cancer.

Currently, EPI and IM are used for the clinical treatment of osteosarcoma (9) and CML (10), respectively. Although the treatment of ovarian cancer has been improving, the treatment effect has been poor due to the prone to chemotherapy resistance of ovarian cancer (11). miR-1301 is related to the anti-tumor effects of EPI and IM (9, 10), and miR-1301 can also inhibit the cisplatin resistance of ovarian cancer through the NF-κB signaling pathway (11). The research progress of miR-1301 can provide new hints for the treatment of osteosarcoma and CML, and also help further study the mechanism of drug resistance in ovarian cancer (**Figure 2**).

### EPI Plays an Anti-Tumor Effect in Osteosarcoma Through the miR-1301/TRIAP1 Axis

EPI has been widely used in the treatment of various cancers, and the resistance of tumors to EPI is related to the enhancement of the bioenergetics of mitochondria (47). TP53-regulated inhibitor of apoptosis 1 (TRIAP1) is overexpressed in many cancers and has been shown to act as an oncogene in tumorigenesis (9). In osteosarcoma, the expression of miR-1301 is down-regulated, while EPI can up-regulate the expression of miR-1301, which can play an anti-osteosarcoma effect by targeting inhibition of TRIAP1 (9).

### IM Exerts an Anti-Tumor Effect in CML Through the miR-1301/RanGAP1 Axis

IM is a 2-phenylaminopyrimidine-based tyrosine kinase inhibitor (48). Although IM is the first-line drug for the treatment of CML at this stage, it is not only expensive but also induces various side effects (10). IM can induce apoptosis of CML cell lines and reduce cell viability. Down-regulation of RanGTPase activating protein 1 (RanGAP1) can enhance the effects of IM (10). By targeting RanGAP1, miR-1301 can disrupt the extranuclear transport of BCR-ABL and induce P73-dependent apoptosis, thereby improving the therapeutic effect of IM for CML (10). The expression of miR-1301 is down-regulated in CML, so the combined use of the ectopic expression of miR-1301 and low-concentration IM can not only achieve a therapeutic effect similar to that of high-concentration IM but also reduce the side effects of IM (10).

### The Molecular Mechanism of miR-1301 and Cisplatin Resistance in Ovarian Cancer

The drug resistance of ovarian cancer can induce tumor cell proliferation and invasion, leading to poor prognosis and even death of patients (11). The expression of miR-1301 is increased in SKOV3/DDP anti-cisplatin ovarian cancer cell line, thereby inhibiting the expression of autophagy genes ATG5 and Beclin1, increasing the expression of EMT-related proteins Snail and Slug, and promoting tumor cell invasion and metastasis (11). miR-1301 can promote autophagy and EMT by activating the NF-κB signaling pathway, while down-regulating miR-1301 can inhibit the NF-κB signaling pathway, thereby preventing the occurrence and development of drug-resistant ovarian cancer (11).

## The miR-1301-Related ceRNA Network

**Figure 3:**
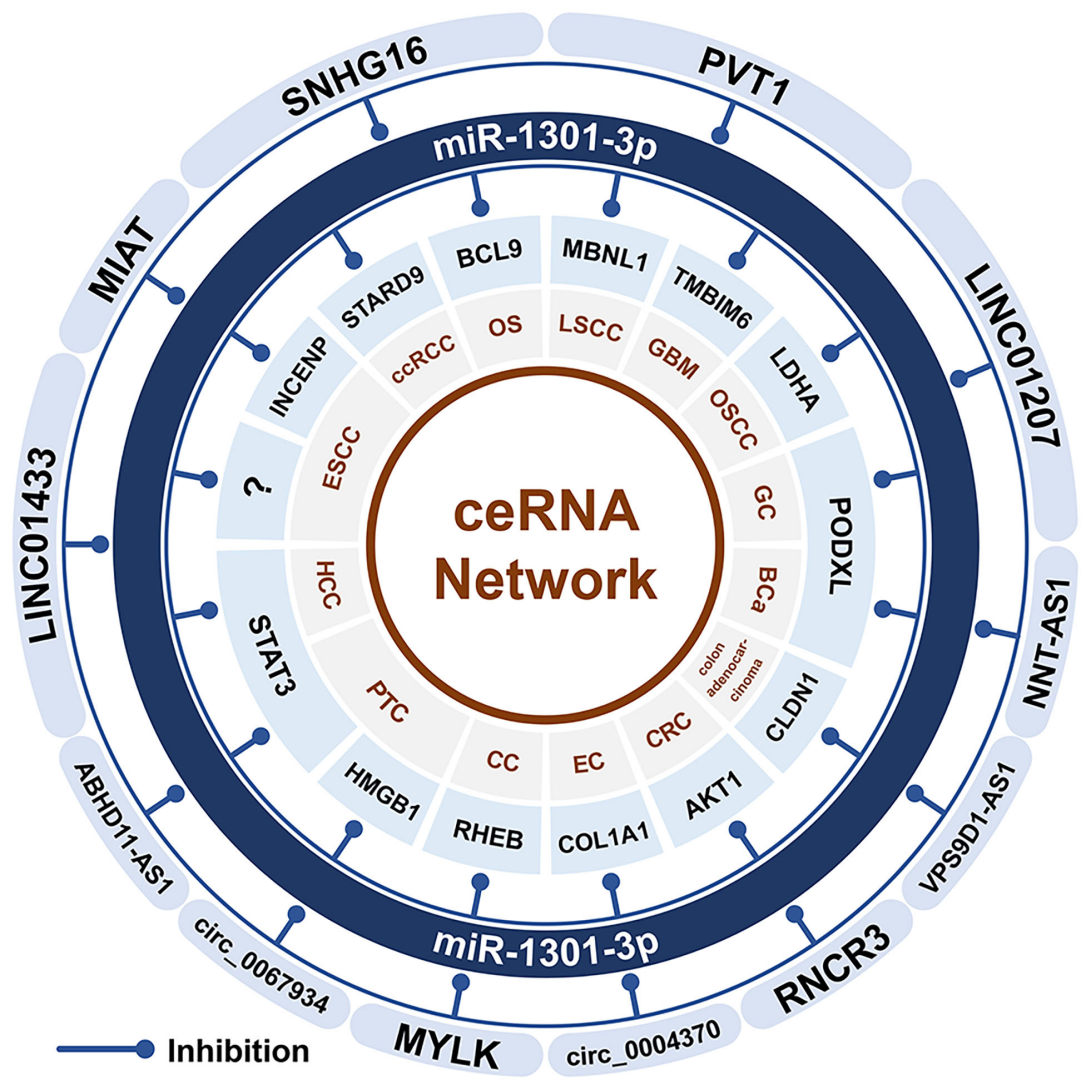
The miR-1301 related ceRNA network. CircRNA and lncRNA can be used as the ceRNA of miR-1301, which promotes the expression of downstream target genes through sponging miR-1301, and participates in the occurrence and development of tumors. GC, gastric cancer; EC, esophageal cancer; ESCC, esophageal squamous cell carcinoma; OSCC, oral squamous cell carcinoma; LSCC, Laryngeal squamous cell carcinoma; CRC, colorectal cancer; GBM, glioblastoma multiforme; CC, cervical cancer; HCC, hepatocellular carcinoma; PTC, papillary thyroid carcinoma; OS, osteosarcoma; ccRCC, clear cell renal cell carcinoma; BCa, bladder cancer.

Both lncRNAs and circRNAs can be used as ceRNAs of miRNAs and participate in the occurrence and development of tumors. The ceRNAs of miR-1301 include at least 3 circRNAs (MYLK and circ_0004370) and 9 lncRNAs (MIAT, LINC01433, RNCR3, LINC01207, ABHD11-AS1, NNT-AS1, SNHG16, and PVT1). All these ceRNAs can cause the down-regulation of miR-1301 expression (**Figure 3**). The miR-1301-related ceRNA network exists in 11 cancers, including cervical cancer (33), esophageal cancer (12, 38, 39), LSCC (41),CRC (17, 18), gastric cancer (1), OSCC (13), PTC (23, 26), bladder cancer (28), ccRCC (27), osteosarcoma (43), and glioblastoma multiforme (GBM) (29).

In cervical cancer, circRNA MYLK up-regulates the expression of RHEB through sponging miR-1301 and promotes tumorigenesis (33). In esophageal cancer, Circ_0004370 up-regulates COL1A1 through sponging miR-1301 to promote the occurrence and development of esophageal cancer (12). In esophageal squamous cell carcinoma (ESCC), SOX2 binds to the MIAT promoter region to promote MIAT expression. Through the MIAT/miR-1301/INCENP axis, MIAT promotes ESCC cell proliferation and cell migration, and invasion (39). Meanwhile, LINC01433 can also sponge miR-1301 to promote the development of ESCC (38). In LSCC cells, NFIB directly binds to the PVT1 promoter region to induce the expression of PVT1, and then the PVT1/miR-1301-3p/MBNL1 axis can promote the proliferation and inhibit apoptosis of LSCC cells (41). Similarly, in CRC, RUNX1 activates RNCR3, and then RNCR3 promotes the occurrence and development of CRC through the RNCR3/miR-1301/AKT1 axis (17). In colon adenocarcinoma, VPS9D1-AS1 can promote tumor cell proliferation and invasion, and inhibit apoptosis through the VPS9D1-AS1/miR-1301-3p/CLDN1 axis (18). In gastric cancer, CTCF activates LINC01207 to promotes proliferation, migration, invasion of cancer cells, and inhibits apoptosis through the LINC01207/miR-1301/PODXL axis (1). In OSCC, LINC01207 can also up-regulate the expression of LDHA by inhibiting miR-1301-3p, thereby promoting the growth and metastasis of OSCC cells (13). In PTC, circ_0067934 upregulates the expression of HMGB1 through sponging miR-1301-3p, thereby promoting the occurrence and development of PTC (23). In addition, STAT3 is the upstream transcription factor of lncRNA ABHD11-AS1. Through STAT3/ABHD11-AS1/miR-1301/STAT3, a feedback loop is formed to down-regulate the expression of miR-1301 in PTC (26). In bladder cancer, NNT-AS1 up-regulates PODXL by sponging miR-1301 (28). In ccRCC, SNHG16 sponges miR-1301 and up-regulates STARD9 (27). SNHG16 expression is up-regulated in osteosarcoma and promotes the occurrence and development of osteosarcoma through the SNHG16/miR-1301/BCL9 axis (43). In addition, in GBM, PVT1 up-regulates TMBIM6 through sponging miR-1301, promotes proliferation and invasion of cancer cells, and inhibits apoptosis (29).

## The miR-1301 Related Signaling Pathways

**Figure 4:**
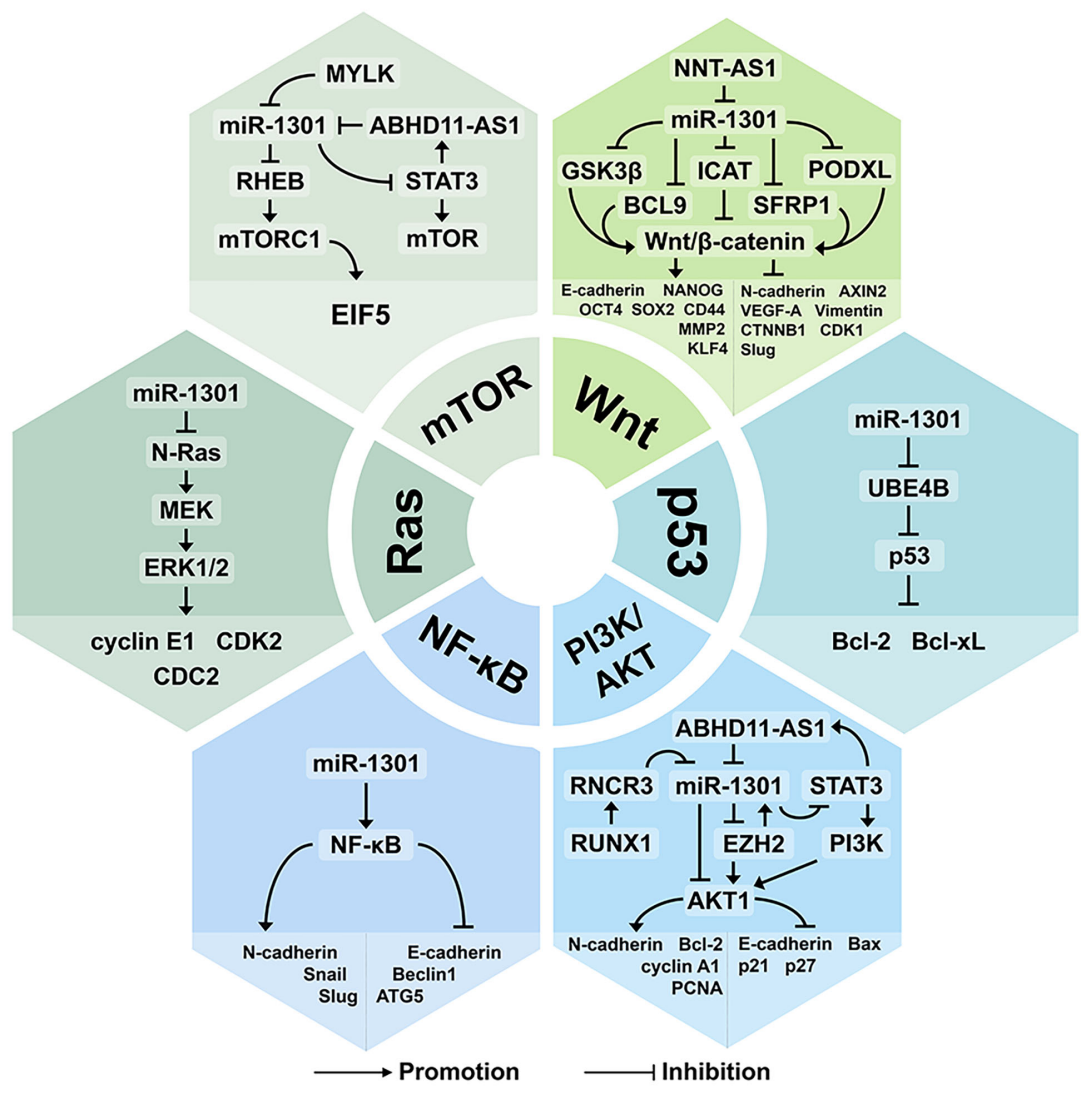
The miR-1301 related signaling pathways. miR-1301 is involved with the Wnt/β-catenin signaling pathway, p53 signaling pathway, mTOR signaling pathway, Ras signaling pathway, NF-κB signaling pathway, and PI3K/AKT signaling pathway.

**Figure 5:**
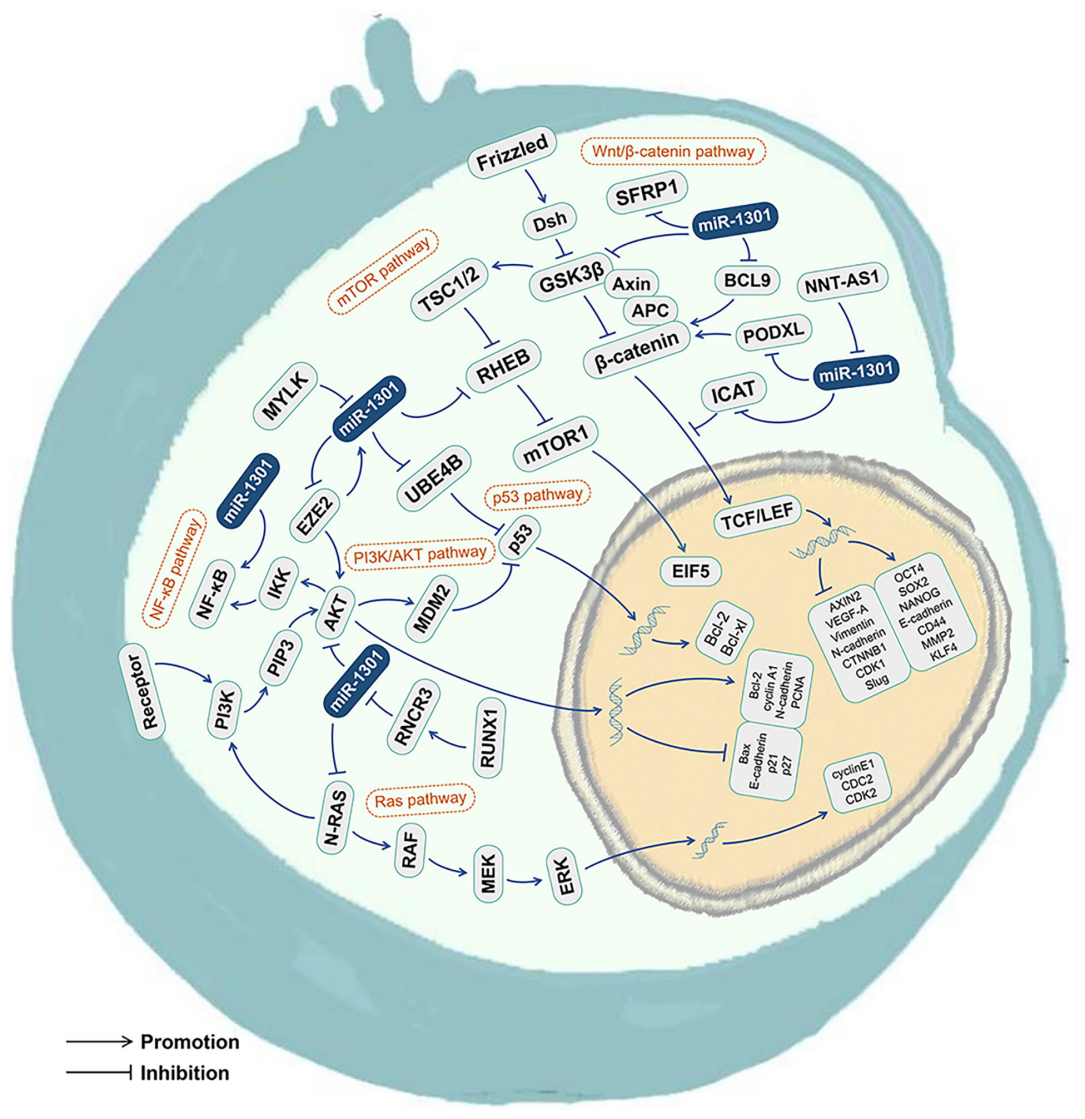
miR-1301 regulates multiple molecular processes in cell.

The tumor growth and development mechanisms involved in miR-1301 include at least 6 classical signaling pathways, including the Wnt/β-catenin signaling pathway (3, 19, 28, 45), p53 signaling pathway (35, 49), mTOR signaling pathway (33), Ras signaling pathway (24), NF-κB signaling pathway (11), and PI3K/AKT signaling pathway (17, 20, 26) (**Figures 4** and **5**, **Table 3**).

### The Wnt/β-Catenin Signaling Pathway

The classic Wnt/β-catenin signaling pathway includes the following key components: WNT, Frizzled, low-density lipoprotein receptor-related protein 5 or 6 (LRP5/6), and β-catenin (50). β-catenin is a signal transduction transcription activator. Mutations of APC, CTNNB1, and other genes can abnormally activate the Wnt/β-catenin signaling pathway, thereby promoting the occurrence and development of a variety of tumors (51). At the same time, the changes in the expression of downstream target genes of the Wnt/β-catenin signaling pathway (such as cyclinD1, MYC, AXIN2, MMP2, MMP9, etc.) may be related to various biological functions of tumor cells, including stemness, proliferation, differentiation, etc. (52).

Many components in the Wnt/β-catenin signaling pathway have become targets for tumor therapy (19). By participating in the Wnt/β-catenin signaling pathway, miR-1301 can regulate the occurrence and development of bladder cancer (28), hepatocellular carcinoma (19), breast cancer (3), and prostate cancer (45).

Up-regulated miR-1301 inhibits PODXL, activates the Wnt/β-catenin signaling pathway, and promotes the growth of bladder cancer cells. Knockdown of lncRNA NNT-AS1 can inhibit the expression of related genes in the Wnt/β-catenin signaling pathway, such as CTNNB1, CDK1, AXIN2, cyclinD1, and MYC (28).

BCL9 is overexpressed in a variety of malignant tumors and can activate the Wnt/β-catenin signaling pathway to promote cell proliferation, migration, and invasion (19). In hepatocellular carcinoma, the down-regulation of miR-1301 expression can up-regulate the expression of its target gene BCL9 and activate the Wnt/β-catenin signaling pathway, thereby inhibiting the migration, invasion, EMT, and angiogenesis of hepatocellular carcinoma (19).

In breast cancer, the expression of miR-1301 is up-regulated and plays the role of an oncogene. ICAT is an important regulator of the Wnt/β-catenin signaling pathway. miR-1301 directly targets and inhibits ICAT, thereby activating the Wnt/β-catenin signaling pathway and promoting the proliferation of breast cancer cells (3).

The up-regulation of miR-1301 in prostate cancer can increase the expression of stem cell-related genes, such as OCT4, SOX2, NANOG, CD44, KLF4, c-MYC, and MMP2. miR-1301 targets the inhibition of GSK3β and SFRP1, thereby activating the Wnt/β-catenin signaling pathway and promoting the proliferation of prostate cancer stem cells (45).

### The p53 Signaling Pathway

p53 acts as a sensor and restricts cell reproduction under high-level pathological conditions, including oncogene signaling, hypoxia, ribosome dysfunction, DNA damage, and nutritional deficiencies. Under low levels of stress, p53 provides pro-survival and protective responses. When cells are exposed to strong stress signals, p53 triggers irreversible senescence or apoptosis programs (53). Ubiquitination factor E4B (UBE4B) is an E3 and E4 ubiquitin ligase that can target phosphorylated p53 and mediate p53 degradation (35). miR-1301 is significantly under-expressed in prostate cancer and can target UBE4B to enhance the function of p53 (49). miR-1301 mediates NO-induced and p53-dependent cell apoptosis by targeting the anti-apoptotic proteins Survivin and MET, thereby participating in colitis-related carcinogenesis (49).

### The mTOR Signaling Pathway

The mammalian target of the rapamycin (mTOR) pathway is involved in many key cellular functions, including protein synthesis, cell cycle progression, apoptosis, and drug resistance (54). mTOR has been widely confirmed as an important downstream molecule of AKT1. The AKT/mTOR signaling pathway mediates tumor metabolic homeostasis and can promote tumor growth and metastasis (33). RHEB is a conserved small GTPase belonging to the Ras superfamily. RHEB is an important upstream regulator of mammalian mTOR signal transduction (33). In cervical cancer, the mTOR signaling pathway is activated and promotes tumorigenesis and development. Inhibition of the MYLK/miR-1301 axis can lead to down-regulation of mTOR pathway-related genes, such as RHEB, EIF5, and mTORC1 (33). In addition, inhibiting the expression of ABHD11-AS1 can increase the levels of p-PI3K, p -AKT, and p-mTOR (26).

### The Ras Signaling Pathway

RAS sarcoma (Ras) protein is a highly homologous small G protein with GTPase activity. RAS sarcoma (Ras) protein is the most frequently activated oncogene in human cancers. N-Ras is involved in cancer and plays a role in the growth, survival, migration, invasion, and angiogenesis of cancer cells (24). In GBM, the expression of miR-1301 is down-regulated, which leads to the activation of the Ras signaling pathway. In glioma cells, overexpression of miR-1301 can down-regulate N-Ras, thereby silencing its downstream MEK-ERK1/2 signaling pathway, inhibiting tumor cell growth, and blocking the G1 phase of the cell cycle (24).

### The NF-κB Signaling Pathway

The NF-κB signaling pathway plays an important role in the processes of autophagy, EMT, cell inflammation, tumor, and immune response (11). NF-κB transcription factor exists in the nucleus and is widely involved in the process of gene transcription (55). The NF-κB family of transcription factors is composed of five different DNA-binding proteins, namely p65 (RELA), p50 (NFKB1), p52 (NFKB2), c-REL, and RELB. They can form up to 15 different homo- or heterodimers (55). The NF-κB signaling pathway is often activated in pathophysiological conditions such as cancer, inflammation, infection, and so on (55). Transfection of cisplatin-resistant strain SKOV3/DDP cell line with miR-1301 mimics can promote the expression of EMT-related genes Snail and Slug, and inhibit the expression of autophagy genes ATG5 and Beclin1. At the same time, it also promotes the expression of NF-κB and N-cadherin and inhibits the expression of E-cadherin. Targeting miR-1301 can inhibit the NF-κB signaling pathway to reduce the proliferation of cisplatin-resistant cells and the development of EMT, thereby preventing the occurrence and development of drug-resistant ovarian cancer (11).

### The PI3K/AKT Signaling Pathway

The PI3K/AKT signaling pathway is one of the most active signaling pathways in human tumors and the main signaling pathway downstream of many growth factor receptors. The PI3K/AKT signaling pathway can phosphorylate PI3K and AKT proteins, thereby promoting tumor cell proliferation and malignant transformation, and inhibiting tumor cell apoptosis (56).

In PTC, ABHD11-AS1 can positively regulate the PI3K/AKT signaling pathway and promote the progression of PTC (26). In TNBC, miR-1301, a downstream gene of EZH2, provides a negative feedback inhibition of EZH2 expression. Meanwhile, miR-1301 increases the levels of p21 and p27 proteins downstream of the PI3K/AKT signaling pathway by reducing the levels of p-AKT and Bcl-XL proteins, thereby playing the role of tumor suppressor genes (20). In CRC, miR-1301 can up-regulate AKT1 to promote the proliferation and invasion of CRC cells, and inhibit cell apoptosis (17).

## Conclusions and Perspectives

miR-1301 is abnormally expressed in 16 cancers and is related to the clinicopathological characteristics of 5 tumors (NSCLC, PTC, CRC, glioma, and osteosarcoma). Besides, miR-1301 may be related to the chemotherapeutic effects of EPI and IM, and participate in the inhibition of cisplatin resistance in ovarian cancer. miR-1301 can interact with 3 circRNAs and 9 lncRNAs in different cancers. In addition, miR-1301 participates in at least 6 classical signaling pathways.

Although miR-1301 is closely related to the occurrence and development of a variety of tumors, the mechanisms that affect various biological processes of tumor cells have not been fully elucidated. First of all, existing studies have shown that miR-1301 acts as a tumor suppressor in 11 types of tumors. However, miR-1301 is a protumor factor in lung cancer (3, 24) and breast cancer (1, 29). The mechanism of these differences has not been clarified yet. Secondly, the expression levels of miR-1301 in hepatocellular carcinoma (23, 41, 42), prostate cancer (20, 21, 43) and gastric cancer (22, 30) tumors are inconsistent. The complex regulation mechanism needs to be further explored in these three cancers. Thirdly, miR-1301 can promote tumor cell metastasis in a variety of tumors, but existing research does not involve the metastasis of cancer cells to secondary sites. In the future, more research on the mechanism of miR-1301 regulating cancer metastasis is needed. Finally, the miR-1301-related ceRNA network described in this work only includes 9 lncRNAs, 3 circRNAs, and 27 mRNAs. Future research needs to focus on discovering more genes and ceRNAs that can interact with miR-1301, which will help further understand the mechanism of miR-1301 in tumors. In addition, the mechanism of miR-1301 in oncogenesis and cancer therapy is also worthy of further research, which will provide a solid theoretical basis for better tumor-targeted therapy and prognosis.

In summary, miR-1301 is a potential tumor target of treatment. More research will focus on the specific molecular mechanism of miR-1301 and its related targets genes in the future. This will lay the foundation for precisely targeted therapy and drug development of related tumors in the future.

## Author Contributions

CZ and YD collected and analyzed literature. CZ drafted the figures. CZ, YD, QZ, CY, and SD wrote this paper. All the authors conceived and gave the final approval of the submitted version.

## Funding

The research was supported by National Natural Science Foundation of China (32100521) and Qiantang Scholar Fund in Zhejiang University City College.

## Conflict of Interest

The authors declare that the research was conducted in the absence of any commercial or financial relationships that could be construed as a potential conflict of interest.

## Publisher’s Note

All claims expressed in this article are solely those of the authors and do not necessarily represent those of their affiliated organizations, or those of the publisher, the editors and the reviewers. Any product that may be evaluated in this article, or claim that may be made by its manufacturer, is not guaranteed or endorsed by the publisher.
